# The German Hemophilia Registry: Growing with Its Tasks

**DOI:** 10.3390/jcm9113408

**Published:** 2020-10-24

**Authors:** Heike Duda, Janina Hesse, Birgit Haschberger, Anneliese Hilger, Christine Keipert

**Affiliations:** 1 dhr Office, Paul-Ehrlich-Institut, Federal Institute for Vaccines and Biomedicines, 63225 Langen, Germany; janina.hesse@pei.de (J.H.); birgit.haschberger@pei.de (B.H.); or dhr@pei.de (C.K.); 2 Division Haematology/Transfusion Medicine, Paul-Ehrlich-Institut, Federal Institute for Vaccines and Biomedicines, 63225 Langen, Germany; anneliese.hilger@pei.de or

**Keywords:** hemophilia, patient registries, gene therapy, EHL clotting factor concentrates, nonreplacement therapies, inhibitors

## Abstract

Hemophilia is a rare heredity bleeding disorder that requires treatment for life. While few therapeutic options were available in the past, multiple recent breakthroughs have fundamentally altered and diversified hemophilia therapy, with even more new therapeutic options forthcoming. These changes are mirrored by significant regulatory and legal changes, which have redefined the role of hemophilia registries in the European Union (EU). This dual paradigm shift poses new regulatory, scientific but also structural requirements for hemophilia registries. The aim of this manuscript is to enumerate these significant challenges and to demonstrate their incorporation into the redesign of the German Hemophilia Registry (Deutsches Hämophilieregister, dhr). To identify the spectrum of hemophilia therapies and the degree of regulatory changes, a horizon screening was performed. Consequently, a core dataset for the dhr was defined by harmonization with regulatory guidelines as well as other hemophilia registries and by heeding the needs of different stakeholders (patients, clinicians, regulators, and scientists). Based on this information, a new registry structure was established, which is optimized for capturing data on new and established hemophilia therapies in a changing therapeutic and regulatory landscape

## 1. Introduction

Hemophilia A (HA) and hemophilia B (HB) are rare heredity bleeding disorders that manifest in frequent and prolonged bleeding episodes and are caused by a lack of blood coagulation factor VIII (FVIII) or factor IX (FIX). Since the discovery of cryoprecipitation by Judith Graham Pool in 1964 [1,2] first allowed for replacement of the missing coagulation factors, steady advances in factor replacement therapy have made hemophilia one of the most treatable rare diseases.

The last decade was a period of rapid innovation in hemophilia therapy. For many years, replacement therapy with human factor products—first plasma-derived, then also recombinant—was the best and only treatment option to manage the symptoms of severe hemophilia. In contrast, during the last ten years, not only did the first recombinant factor products with extended plasma half-life (EHL products) reach marketing authorization, but also, the first non-factor-based product for hemophilia treatment was authorized for use in the EU, signaling a paradigm shift in hemophilia therapy. Several more promising products, both factor-based and non-factor-based, are currently undergoing clinical trials. And all of these changes may be eclipsed by the introduction of gene therapy, with its tantalizing promise of a functional cure for hemophilia.

While these new therapies are a source of hope for people with hemophilia (PWH) and their families, each of them also poses new and unique challenges for the regulatory authorities tasked with ensuring safety and efficacy of the products. In the rapidly evolving landscape of hemophilia treatment, valid and meaningful observational data on the epidemiology and treatment of hemophilia are becoming increasingly important. The obvious choice for collecting these data are hemophilia registries.

National hemophilia registries already exist in most European countries [3,4] and data on hemophilia incidence are collected in many countries worldwide [5]. Hemophilia is a rare disease with an expected number of 1,125,000 people with hemophilia (PWH) worldwide (418,000 of whom with severe hemophilia [6]) many of whom may remain undiagnosed [5] and therefore not be included in registries. Thus, data from one country may not be sufficient to obtain conclusive results. Due to the scarcity of eligible patients, clinical trials on products for the treatment of hemophilia are already routinely conducted internationally. Following this example, there are several initiatives to harmonize data collection in hemophilia registries and to advance hemophilia research through international cooperation. An ambitious project is the World Bleeding Disorders Registry (WBDR), which was established by the World Federation of Hemophilia (WFH) in 2018 to become a new global hemophilia registry [7]. In the EU, the Patient Registry Initiative—launched by European Medicines Agency (EMA) in 2015 [8]—promotes the harmonization of patient registries [9]. Additionally, several workshops were initialized to analyze the possibility to include existing registries and registry data in regulatory processes [10,11]. In the United Kingdom, the National Haemophilia Database (NHD) has since 2008 successfully conducted several EMA-mandated post-marketing studies [12,13], demonstrating the potential value of well-defined and well-managed hemophilia registries for the regulatory authorities. Accordingly, hemophilia was one of the first indications to be included in the patient registry initiative [10]. The marketing authorization requirements of relevant medicinal products were subsequently updated when the 2nd revision of the Guideline on the clinical investigation of recombinant and human plasma-derived factor VIII products came into effect in 2019 [11]. The updated guideline not only strengthens the role of hemophilia registries by suggesting all PWH should be included in registries, but also sets the framework for the inclusion of these hemophilia registries in post-marketing studies. Another topic addressed by the revised guideline is the harmonization of existing national and international hemophilia registries. While hemophilia registries can and do collaborate in regulatory and scientific projects (e.g., [14]), the opportunities for collaboration are limited to registries with compatible datasets. By providing a core dataset for well-defined hemophilia registries in the revised guideline [11], EMA seeks to spur harmonization and facilitate future collaborations between European hemophilia registries.

In Germany, these regulatory changes on EU level were supplemented by an update of the German Transfusion Act (Transfusionsgesetz, TFG). Like many European countries, Germany already had a national hemophilia registry: The German Hemophilia Registry (Deutsches Hämophilieregister, dhr), which was tasked with collecting the data of PWH and other people with inherited bleeding disorders in Germany. The first iteration of the dhr—a web portal maintained by the Paul-Ehrlich-Institut (PEI)—was established in 2008 as a cooperation project between the Society of Thrombosis and Hemostasis Research (Gesellschaft für Thrombose- und Hämostaseforschung, GTH), the two patient advocacy organizations (Deutsche Hämophiliegesellschaft zur Bekämpfung von Blutungskrankheiten e.V., DHG and Interessengemeinschaft Hämophiler e.V., IGH) and the PEI as the responsible federal authority [15,16]. Participation in the dhr is mandatory for hospitals, medical practices and other medical facilities treating people with inherited bleeding disorders (hereinafter referred to as “centers”). In 2018, 4240 people with HA and 785 people with HB were included in the dhr, 2583 and 403 of whom had severe hemophilia [17]. Assuming an average prevalence of severe hemophilia (see [6]) and a German population of 83 million, these patient numbers equal 104% and 88% of expected cases of severe HA or HB in Germany. Before the amendment of the TFG—effective in 2019—, the primary objective of the dhr was to capture the supply situation with medicinal products for the treatment of inherited bleeding disorders and thus provide data for supply planning. The amendment of the TFG expanded the statutory tasks of the dhr to also include collaboration with other hemophilia registries and the collection of data suitable for research projects [18,19]. It quickly became apparent that to capture these data in the dhr—previously primarily optimized to capture factor consumption data—a major redesign of the registry was required.

To map the recent changes in the landscape of hemophilia treatment, a horizon screening was performed to identify (1) new and upcoming therapeutic options for hemophilia therapy; (2) regulatory changes on national, European and international level; and (3) the needs/demands of the stakeholders involved in the dhr: Patients, clinicians, regulators and scientists. The new dhr was then established based on these findings.

The aim of this manuscript is to describe how the recent challenges for hemophilia registries were identified and addressed in the redesign of the dhr in 2019 to enable the registry not only to collect clinical data, but also to meet the recent and future challenges in hemophilia therapy.

## 2. Experimental Section

### 2.1. Assessment of Current dhr

The current set-up of the dhr was analyzed and essential structures were identified for transfer to the new system.

### 2.2. Horizon Screening: Scientific and Regulatory Environment of Hemophilia Therapy

To identify products and product classes likely to become relevant for treatment of hemophilia and other bleeding disorders in the near future, clinicaltrials.gov was searched for current and planned clinical trials with the keyword “hemophilia”. Special consideration was given to non-factor-based products undergoing clinical trials for hemophilia treatment and to all products in phase III clinical trials at the time of screening.

The regulatory environment for clinical trials for marketing authorization and post-marketing surveillance of medicinal products for treatment of hemophilia was assessed and relevant laws and guidelines on both German and European level were identified. If no regulation on a relevant topic was available on either level, regulatory documents from other countries were assessed for guidance (e.g., Food and Drug Administration (FDA) guidance on hemophilia gene therapy [20]).

Publications about other national and international hemophilia registries were identified by PubMed searches for “hemophilia registry” and variants and by name search of known registries. Datasets and protocols were obtained from official reports or public websites of these other hemophilia registries (e.g., National Hemophilia Database (NHD): [21,22] Australian Bleeding Disorders Registry (ABDR): [23] Réseau FranceCoag (FranceCoag): [24,25,26] PedNet: [27] GEPHARD: [28])

### 2.3. New dhr Dataset

Based on this information, a new dataset for the dhr was developed. The main considerations were the ability to include new products (both factor- and non-factor-based) and gene therapy, compliance with regulatory requirements on national and international level, harmonization with other registries and with the core dataset of the EMA, scientific relevance, and ease of use. The complete new dataset of the dhr can, for reasons of transparency and harmonization, be downloaded from the homepage of the dhr under “DHR-Service” [29].

## 3. Results

### 3.1. Inclusion of New Hemophilia Therapies

In recent years, several new therapies for treatment of hemophilia—both factor- and non-factor-based—have received marketing authorization, while more are undergoing clinical trials [30,31] In novel factor products, so-called extended half-life (EHL) products, plasma half-life was prolonged up to 1.5-fold for FVIII and 2.4–4.8-fold for FIX by modification of recombinant FVIII and FIX proteins [32]. EHL products were first introduced to the German market in 2016 (FIX, Alprolix [33] and Idelvion [34]). Also, the first non-factor-based medicinal product was authorized for the European market for treatment of HA patients with FVIII inhibitors in 2018 with Emicizumab (Hemlibra) [35], a bispecific monoclonal antibody which mimics FVIII function [36,37]. Other non-factor products undergoing clinical trials include Fitusiran, a small interfering RNA (siRNA) that blocks the synthesis of the blood coagulation inhibitor Antithrombin [38,39,40], and the monoclonal antibodies Concizumab [41,42] and Marstacimab (PF-06741086) [43], which target TFPI, an inhibitor of the tissue factor pathway [44,45]. To facilitate data capture for new therapies, EHL products and Emicizumab were included as separate classes of therapeutics in the dhr. As many new hemophilia therapies are not yet authorized for the German market, patients can be reported as participating (or having participated) in a given clinical trial. For this purpose, each class of therapeutics in the dhr includes the option “Studienpräparat” (investigational medicinal product), which can be used to report consumption data without disclosing which product was consumed. Last, provisions were made to allow for inclusion of additional therapies in the dhr upon their marketing authorization.

Another promising therapeutic approach is hemophilia gene therapy. Unlike replacement therapy, a successful gene therapy is a one-time treatment, requiring a vastly different dataset when included in a registry. While gene therapy, as to yet, has not been authorized for treatment of hemophilia in the EU, clinicaltrials.gov currently (September 2020) lists seven phase 3 clinical trials investigating five different potential hemophilia gene therapies. Investigational medicinal products include Valoctocogene Roxaparvovec (previously BMN 270) [46,47,48] and PF-07055480 [49] for treatment of HA and Fidanacogene Elaparvovec (previously PF-06838435) [50], FLT180a [51], and AMT-061 [52] for treatment of HB. As the WFH Gene Therapy Round Table suggests recording gene therapy for hemophilia in central or national registries [53,54], considering upcoming gene therapies in the redesign of the dhr seemed prudent. To harmonize clinical trials on hemophilia gene therapy in the jurisdiction of the United States, the Food and Drug Administration (FDA) issued the Guidance “Human Gene Therapy for Hemophilia” [20], which has influenced the design of many clinical trials since the publication of its first draft in 2018. In addition, clinical trials for hemophilia gene therapies were screened to identify other recurring outcome measures, supplemental treatments and relevant inclusion and exclusions criteria. Including these parameters, the dhr also covers most relevant outcome measures of the CoreHEM dataset, which was suggested for registering gene therapy by the coreHEM multistakeholder project after the dataset of the dhr 2.0 was finalized [55]. With the addition of the gene therapy module, the dhr is one of the first national hemophilia registry able to cover gene therapy in its dataset.

### 3.2. Harmonization with EMA Guidelines and Existing Hemophilia Registries

When the 2nd revision of the European Medicines Agency (EMA)’s “*Guideline on the clinical investigation of recombinant and human plasma-derived factor VIII products*” came into effect in 2019, it fundamentally changed the marketing authorization requirements for FVIII products. While in revision 1 of the guideline, clinical trials in both previously treated patients (PTPs) and previously untreated patients (PUPs) were required [56], revision 2 no longer requires separate clinical trials in PUPs before use of product in this patient group, but strongly suggests all PUPs and PTPs should be monitored in well-defined and well-managed disease registries [11]. A similarly worded revision of EMA “*Guideline on the clinical investigation of recombinant and human plasma-derived factor IX products*” is currently in progress [57]. This change in policy strengthens the role of hemophilia registries in post-marketing surveillance of factor products in the European Union (EU) and enhances the need for the harmonization of the existing European hemophilia registries—which is a prerequisite for this role. At the same time, the revised guidelines propose an essential core dataset for hemophilia registries [11,57], which was implemented also in the dhr.

The primary objective of the previous dhr was to capture the supply situation with medicinal products for the treatment of inherited bleeding disorders. Also in 2019, changes in the Transfusion Act (Transfusionsgesetz, TFG) expanded the statutory tasks of the dhr on the national level: It was also tasked (1) to collect and provide the necessary data to promote research about inherited bleeding disorders and (2) to improve international cooperation with other hemophilia registries [18,19]. These tasks especially necessitated the redesign of the registry, as they require increased capture of clinical and research data (1) and harmonization of the dataset with other hemophilia registries (2). For further harmonization with other registries and to ensure the relevance and completeness of the collected data, the datasets of several hemophilia registries (National Hemophilia Database (UK), FranceCoag (France), and the international and German PUP registries PedNet and GEPHARD) were taken into account to identify parameter candidates for expansion of the dhr dataset. The complete new dataset is available on the homepage of the dhr under “DHR-Service” [29]. Also, parameter definitions in the new dhr were harmonized with other registries and with the literature wherever possible to increase compatibility of captured data with other registries.

The transformation of the existing dhr into a hemophilia registry suited to these new regulatory challenges did not only require an expansion of the dataset, but also the inclusion of new patient subgroups with specialized data capture needs. A subgroup especially relevant to post-marketing surveillance are PUPs, as with the end of mandatory clinical trials in PUPs with hemophilia, post-marketing data captured in hemophilia registries will become increasingly important to optimize hemophilia treatment for PUPs as compared to PTPs. One major difference between PUPs and PTPs is inhibitor risk, which is highest in PUPs just beginning treatment and much lower in PTPs [58,59,60,61,62,63]. To ensure relevance and completeness of patient data captured in this early stage of treatment, the dhr was harmonized with the designated PUP registries PedNet and GEPHARD. Other new patient subgroups in the dhr are patients with von-Willebrand disease (vWD) type 3 and patients with rare inherited bleeding disorders caused by deficiency of coagulation factors I, II, V, VII, X, XI, or XIII, whose prevalence in Germany will be comprehensively captured in the new dhr for the first time.

### 3.3. Compatibility with Previous dhr

One of the biggest advantages of well-managed disease registries is that unlike clinical trials, the lifespan of a registry is not limited by design—as exemplified by the NHD, established in 1968 [22]. This makes it possible to capture longitudinal treatment data far beyond the scope of most clinical studies—data which become more valuable for research with every additional year. The first iteration of the dhr has been collecting factor consumption and treatment data from 2008–2019. It was therefore a priority to transfer these data to the new registry, to ensure continuity of data capture between the old and the new registry, and to ensure the compatibility of data captured in different iterations of the dhr. To do so, the datasets of the old and new dhr were harmonized and all important parameters in the old dhr were carefully matched to parameters in the new registry. Also, the data model of the new dhr was structured after its previous iteration, and central data protection measures of the old registry were maintained in the new registry.

The dhr is, by design, a hybrid between epidemiological and clinical registry. Its mode of data capture therefore needs to reflect both epidemiological and clinical questions while respecting patients’ right to informational self-determination. While all patients are encouraged to participate, single data capture is a strictly opt-in program and requires the informed consent of the patient. These donated data are then stored in the dhr in pseudonymized form, precluding the reidentification of the patient. The personal information of patients and consent information is managed locally at the participating centers, and participating patients are known to the dhr staff only by their registry IDs. For patients who did not volunteer to participate in the dhr, only minimal anonymous data are captured for supply planning in a collective report, as mandated by the Transfusion Act (TFG § 21 (1a)) [18].

The dataset of single data capture contains profile data, diagnostic data and treatment data. Profile data consists of few demographic markers (e.g., year of birth) captured to allow for formation of patient subgroups. Diagnostic data refers to the patient’s diagnosis and treatment history (e.g., severity of diagnosis and date of first bleeding event). Both are collected once upon registration of a new patient to establish a data baseline. Therapeutic data refers to current health and treatment of the patient (e.g., factor consumption, recent inhibitor tests). As such, it needs to be reported at least annually. This data structure remains mostly unchanged, even as the dataset of the new dhr became more comprehensive and complex in the course of the redesign.

Like the previous dhr, the new registry is a web portal solution, into which data can be entered manually by registered users at the participating centers. Parameters are organized in modules, some of which are universal, while others are shown in a context-dependent manner (e.g., only for a certain diagnosis) to facilitate handling of the larger dataset. Modular and context-dependent data capture—already present in the first iteration of the dhr—was expanded in the new dhr to reduce the workload for the participating centers. In addition, an extensive help function and detailed manuals are provided with the new registry. To facilitate data capture further, in the previous dhr an interface was implemented which enabled centers to synchronize data from home therapy diaries into the dhr. It is planned to reinstate a comparable interface solution in the new dhr.

### 3.4. A New German Hemophilia Registry

Based on the requirements outlined above, a new dhr was designed and established in 2018–2019. The new system launched in 2019 for selected patient subgroups first and will collect the data of all German patients from the annual reports 2020 onwards.

## 4. Discussion

With the launch of the new dhr for all patient groups in 2020, the redesign of the dhr into a registry tailored to the current and future challenges in hemophilia therapy will conclude—for now. In the redesign of the dhr, multiple challenges faced by hemophilia registries today were identified and addressed to create a state of the art new registry. But as new therapies and new regulatory requirements continue to reshape the landscape of hemophilia therapy, these challenges are bound to resurface in the future. In a changing treatment landscape, the continued ability of the dhr to adapt to new therapeutic and regulatory situations will be central for the future success of the registry.

In a horizon screening, new therapies and therapeutic approaches were identified and preemptively included in the new dhr. These include new therapies such as gene therapy, which requires a vastly different approach to data capture than replacement therapy. In case of future therapeutic changes, the data model of the new registry is also flexible enough to include more parameters and therapies at will. This is required because the new dhr dataset, though designed with the therapies of tomorrow in mind, is itself only a snapshot of hemophilia treatment. In a rapidly changing field such as hemophilia therapy, the flexible design of the registry is a prerequisite to ensure continued relevance of the collected data. Another prerequisite is the early identification of relevant future therapies in scientific publications and the clinical trial pipeline and their timely and concise inclusion in the registry before marketing authorization—a task for which strong ties to the regulatory authorities and to the scientific and medical community will be vital. The most important prerequisite for continued relevance of the dhr, though, will be the continued inclusion of all dhr stakeholders—patients, clinicians, regulators and scientists—to ensure that even in changing times, the dhr keeps serving the needs of the German hemophilia community.

The new dhr was designed to capture not only new, but also existing hemophilia therapies at single-patient resolution. For the capture of clinical data in the dhr, a core dataset for existing hemophilia therapies was identified and incorporated. This dataset both fulfills the requirements of the EMA guidelines for post-marketing monitoring in hemophilia registries, and allows for harmonized capture of research data—as mandated by the Transfusion Act—by extensive harmonization with other hemophilia registries. The dataset and data structure of the new dhr were carefully harmonized with its previous iteration as well, and the data collected in the last twelve years were transferred to the new registry for future research projects. This new framework not only increases the scientific relevance of the captured data during one of the most interesting transitions in hemophilia treatment, but will also enable the dhr to collect valuable real-world information for the benefit-risk evaluation of medicinal products. For a disease as rare as hemophilia, international cooperation and harmonization of efforts between different institutions will only become more important in the future. The EMA heeded this development by the formal inclusion of registries in post-authorization data collection in the EU. This not only allows for the use of registry data to identify new and emerging safety information in the post-marketing phase (summarized in Periodic Safety Update Reports, PSURs), but also is an opportunity to conduct post-marketing studies of established and new therapies directly in and in collaboration with European hemophilia registries. With this decision, the first steps were made to formally include registries into the regulatory process on a European level and to broaden the pool of data available to inform regulatory decisions on product safety and efficacy.

Besides its expected benefits for patient safety, this new regulatory framework also has the potential to advance hemophilia research in Europe by fostering collaborations between registries. As in other rare diseases, availability of data is a limiting factor in hemophilia research—a limiting factor that can be remedied by international collaboration and sharing of research data. While individual hemophilia registries already do collaborate with each other, the modalities of merging registry data should be defined on a European level to take full advantage of synergies between different European hemophilia registries in future regulatory and scientific projects. By gathering available data currently dispersed over various EU countries, European collaboration of hemophilia registries will hopefully contribute to improving safety and efficacy of established therapies and facilitate research on established but also new therapeutic approaches—developments which in turn will benefit the PWH in the EU. Because judging from the advances of the past decade, the future of hemophilia treatment may have only just begun.

## References

[1] Pool J.G., Shannon E.A. (1965). Production of high-potency concentrates of antihemophilic globulin in a closed-bag system. N. Engl. J. Med..

[2] Knight T., Callaghan M.U. (2018). The role of emicizumab, a bispecific factor IXa- and factor X-directed antibody, for the prevention of bleeding episodes in patients with hemophilia A. Ther. Adv. Haematol..

[3] Keipert C., Hesse J., Haschberger B., Heiden M., Seitz R., van den Berg H.M. (2015). The growing number of hemophilia registries: Quantity vs. quality. Clin. Pharmacol. Ther..

[4] Mahony B.O., Savini L., Hara J.O., Bok A. (2017). Haemophilia care in Europe—A survey of 37 countries. Haemoph. Off. J. World Fed. Hemoph..

[5] Stonebraker J.S., Bolton-Maggs P.H., Brooker M., Evatt B., Iorio A., Makris M., Tootoonchian E. (2020). The World Federation of Hemophilia Annual Global Survey 1999–2018. Haemoph. Off. J. World Fed. Hemoph..

[6] Iorio A., Stonebraker J.S., Chambost H., Makris M., Coffin D., Herr C., Germini F. (2019). Establishing the Prevalence and Prevalence at Birth of Hemophilia in Males: A Meta-analytic Approach Using National Registries. Ann. Intern. Med..

[7] World Bleeding Disorders Registry (2018). World Bleeding Disorders Registry: 2018 Data Report. https://www.wfh.org/en/our-work-research-data/world-bleeding-disorders-registry.

[8] European Medicines Agency (2015). Initiative for Patient Registries: Strategy and Pilot Phase. https://www.ema.europa.eu/documents/other/initiative-patient-registries-strategy-pilot-phase_en.pdf.

[9] European Medicines Agency (2017). Patient Registry Initiative—Strategy and Mandate of the Cross-Committee Task Force. https://www.ema.europa.eu/documents/other/patient-registry-initiative-strategy-mandate-cross-committee-task-force_en.pdf.

[10] European Medicines Agency (2018). Report on Haemophilia Registries—Workshop 8 June 2018. https://www.ema.europa.eu/documents/report/report-haemophilia-registries-workshop_en.pdf.

[11] European Medicines Agency Guideline on the Clinical Investigation of Recombinant and Plasma-Derived FVIII Products: EMA/CHMP/BPWP/144533/2009 rev. 2, 2018. https://www.ema.europa.eu/en/clinical-investigation-recombinant-human-plasma-derived-factor-viii-products.

[12] Hay C.R.M., Sharpe T., Dolan G. (2017). Use of the UKHCDO Database for a postmarketing surveillance study of different doses of recombinant factor VIIa in haemophilia. Haemoph. Off. J. World Fed. Hemoph..

[13] Mathias M.C., Collins P.W., Palmer B.P., Chalmers E., Alamelu J., Richards M., Will A., Hey C.R.M., United Kingdom Haemophilia Centre Doctors’ Organisation Inhibitor Working Party (2018). The immunogenicity of ReFacto AF (moroctocog alfa AF-CC) in previously untreated patients with haemophilia A in the United Kingdom. Haemoph. Off. J. World Fed. Hemoph..

[14] Volkers P., Hanschmann K.M., Calvez T., Chambost H., Collins P.W., Demiguel V., Palmer B.P. (2019). Recombinant factor VIII products and inhibitor development in previously untreated patients with severe haemophilia A: Combined analysis of three studies. Haemoph. Off. J. World Fed. Hemoph..

[15] Haschberger B., Hesse J., Heiden M., Seitz R., Schramm W. (2010). Dokumentation in der Hämophilietherapie mit Unterstützung des Deutschen Hämophilieregisters. Hämostaseologie.

[16] Haschberger B., Hesse J., Heiden M., Seitz R., Schramm W. (2009). DHR—Ready for take-off. Hämostaseologie.

[17] Geschäftsstelle des Deutschen Hämophilieregisters (dhr office) Patientenzahlen: Grafiken seit 2012. https://www.pei.de/SharedDocs/Downloads/DE/regulation/meldung/dhr-deutsches-haemophilieregister/meldung-21-tfg-tabellen-grafiken.xlsx?__blob=publicationFile&v=7.

[18] Gesetz zur Regelung des Transfusionswesens (Transfusionsgesetz): TFG. https://www.gesetze-im-internet.de/tfg/index.html.

[19] Gesetz zur Fortschreibung der Vorschriften für Blut- und Gewebezubereitungen und zur Änderung anderer Vorschriften: GSAV, 2017. https://www.bgbl.de/xaver/bgbl/start.xav#__bgbl__%2F%2F*%5B%40attr_id%3D%27I_2017_52_inhaltsverz%27%5D__1600334071659.

[20] Human Gene Therapy for Hemophilia; Guidance for Industry: FDA 2018-D-2238; 2020. https://www.fda.gov/regulatory-information/search-fda-guidance-documents/human-gene-therapy-hemophilia.

[21] National Haemophilia Database National Haemophilia Database Dataset: Data Set 2018. http://www.ukhcdo.org/nhd/.

[22] Dolan G., Makris M., Bolton-Maggs P.H.B., Rowell J.A. (2014). Enhancing haemophilia care through registries. Haemoph. Off. J. World Fed. Hemoph..

[23] National Blood Authority ABDR User Manual. V 1.11, October 2015. https://www.blood.gov.au/abdr.

[24] Réseau FranceCoag (2016). Protocole V2 du Réseau FranceCoag. http://www.francecoag.org/SiteWebPublic/html/documentsTele.html.

[25] Calvez T., Biou M., Costagliola D., Jullien A.M., Laurian Y., Rossi F. (2001). The French haemophilia cohort: Rationale and organization of a long-term national pharmacosurveillance system. Haemoph. Off. J. World Fed. Hemoph..

[26] Réseau FranceCoag Formulaires: Cohorte Générale, Sous-Cohorte PUPs, Autres. http://www.francecoag.org/SiteWebPublic/html/documentsTele.html.

[27] Fischer K., Ljung R., Platokouki H., Liesner R., Claeyssens S., Smink E., Van den Berg H.M. (2014). Prospective observational cohort studies for studying rare diseases: The European PedNet Haemophilia Registry. Haemoph. Off. J. World Fed. Hemoph..

[28] Ludwig-Maximilians-University of Munich (Sponsor), Pediatric Committee of the German Thrombosis and Hemostasis Research Society (Collaborator), Society of Thrombosis and Haemostasis Research (Germany) (Collaborator), and C. Bidlingmaier (Principal Investigator), German Pediatric Hemophilia Research Database (GEPHARD). NCT02912143.

[29] Geschäftsstelle des Deutschen Hämophilieregisters (dhr Office), Gesamtdatensatz dhr. https://www.pei.de/DE/regulation/melden/dhr/dhr-node.html/?cms_tabcounter=5.

[30] Weyand A.C., Pipe S.W. (2019). New therapies for hemophilia. Blood.

[31] Miesbach W., Schwäble J., Müller M.M., Seifried E. (2019). Treatment Options in Hemophilia. Dtsch. Ärzteblatt Int..

[32] Ar M.C., Balkan C., Kavaklı K. (2019). Extended Half-Life Coagulation Factors: A New Era in the Management of Hemophilia Patients. Turk. J. Haematol. Off. J. Turk. Soc. Haematol..

[33] European Medicines Agency European Public Assessment Report—Alprolix. https://www.ema.europa.eu/en/medicines/human/EPAR/alprolix.

[34] European Medicines Agency European Public Assessment Report—Idelvion. https://www.ema.europa.eu/en/medicines/human/EPAR/idelvion.

[35] European Medicines Agency European Public Assessment Report—Hemlibra. https://www.ema.europa.eu/en/medicines/human/EPAR/hemlibra.

[36] Sampei Z., Igawa T., Soeda T., Okuyama-Nishida Y., Moriyama C., Wakabayashi T., Yoshihashi K. (2013). Identification and multidimensional optimization of an asymmetric bispecific IgG antibody mimicking the function of factor VIII cofactor activity. PLoS ONE.

[37] Lenting P.J., Denis C.V., Christophe O.D. (2017). Emicizumab, a bispecific antibody recognizing coagulation factors IX and X: How does it actually compare to factor VIII?. Blood.

[38] Machin N., Ragni M.V. (2018). An investigational RNAi therapeutic targeting antithrombin for the treatment of hemophilia A and B. J. Blood Med..

[39] Genzyme, a Sanofi Company (Sponsor, Investigator) A Study of Fitusiran (ALN-AT3SC) in Severe Hemophilia A and B Patients with Inhibitors (ATLAS-INH). NCT03417102.

[40] Genzyme, a Sanofi Company (Sponsor, Investigator) A Study of Fitusiran (ALN-AT3SC) in Severe Hemophilia A and B Patients without Inhibitors. NCT03417245.

[41] Novo Nordisk A/S (Sponsor, Investigator) Research Study to Look at How Well the Drug Concizumab Works in Your Body if You Have Haemophilia without Inhibitors (explorer8). NCT04082429.

[42] Novo Nordisk A/S (Sponsor, Investigator) Research Study to Look at How Well the Drug Concizumab Works in Your Body if You Have Haemophilia with Inhibitors (explorer7). NCT04083781.

[43] Pfizer (Sponsor, Investigator) Study of the Efficacy and Safety PF-06741086 in Adult and Teenage Patients with Severe Hemophilia A or B. NCT03938792.

[44] Chowdary P. (2020). Anti-tissue factor pathway inhibitor (TFPI) therapy: A novel approach to the treatment of haemophilia. Int. J. Hematol..

[45] Patel-Hett S., Martin E.J., Mohammed B.M., Rakhe S., Sun P., Barrett J.C., Brophy D.F. (2019). Marstacimab, a tissue factor pathway inhibitor neutralizing antibody, improves coagulation parameters of ex vivo dosed haemophilic blood and plasmas. Haemoph. Off. J. World Fed. Hemoph..

[46] BioMarin Pharmaceutical (Sponsor, Investigator) Single-Arm Study to Evaluate the Efficacy and Safety of Valoctocogene Roxaparvovec in Hemophilia a Patients (BMN 270-301). NCT03370913.

[47] BioMarin Pharmaceutical (Sponsor, Investigator) Single-Arm Study to Evaluate the Efficacy and Safety of Valoctocogene Roxaparvovec in Hemophilia a Patients at a Dose of 4E13 vg/kg (BMN270-302). NCT03392974.

[48] BioMarin Pharmaceutical (Sponsor, Investigator) Study to Evaluate the Efficacy and Safety of Valoctocogene Roxaparvovec, with Prophylactic Steroids in Hemophilia a (GENEr8-3). NCT04323098.

[49] Pfizer (Sponsor, Investigator) Study to Evaluate the Efficacy and Safety of PF-07055480 in Moderately Severe to Severe Hemophilia a Adults (AFFINE). NCT04370054.

[50] Pfizer (Sponsor, Investigator) A Study to Evaluate the Efficacy and Safety of Factor IX Gene Therapy with PF-06838435 in Adult Males With Moderately Severe to Severe Hemophilia B (BENEGENE-2). NCT03861273.

[51] Freeline Therapeutics (sponsor) A Long-Term Follow-Up Study of Haemophilia B Patients Who Have Undergone Gene Therapy. NCT03641703.

[52] UniQure Biopharma B.V., Pipe S. HOPE-B: Trial of AMT-061 in Severe or Moderately Severe Hemophilia B Patients. NCT03569891.

[53] Pierce G.F., Coffin D. (2019). The 1st WFH Gene Therapy Round Table: Understanding the landscape and challenges of gene therapy for haemophilia around the world. Haemoph. Off. J. World Fed. Hemoph..

[54] Pierce G.F., Pasi K.J., Coffin D., Kaczmarek R., Lillicrap D., Mahlangu J., Weill A. (2020). Towards a global multidisciplinary consensus framework on haemophilia gene therapy: Report of the 2nd World Federation of Haemophilia Gene Therapy Round Table. Haemoph. Off. J. World Fed. Hemoph..

[55] Iorio A., Skinner M.W., Clearfield E., Messner D., Pierce G.F., Witkop M., Pezalla E. (2018). Core outcome set for gene therapy in haemophilia: Results of the coreHEM multistakeholder project. Haemoph. Off. J. World Fed. Hemoph..

[56] European Medicines Agency Guideline on the Clinical Investigation of Recombinant and Human Plasma-Derived Factor VIII Products: EMA/CHMP/BPWP/144533/2009 rev. 1, 2016. https://www.ema.europa.eu/en/clinical-investigation-recombinant-human-plasma-derived-factor-viii-products.

[57] Guideline on Clinical Investigation of Recombinant and Human Plasma-Derived Factor IX Products: EMA/CHMP/BPWP/144552/2009 rev. 2 Corr.1, 2018. https://www.ema.europa.eu/en/clinical-investigation-recombinant-human-plasma-derived-factor-ix-products.

[58] Van Den Berg H.M., Fischer K., Carcao M., Chambost H., Kenet G., Kurnik K., Ljung R. (2019). Timing of inhibitor development in more than 1000 previously untreated patients with severe hemophilia A. Blood.

[59] Male C., Andersson N.G., Rafowicz A., Liesner R., Kurnik K., Fischer K., Königs C. (2020). Inhibitor incidence in an unselected cohort of previously untreated patients with severe haemophilia B: A PedNet study. Haematologica.

[60] Hassan S., Cannavò A., Gouw S.C., Rosendaal F.R., van der Bom J.G. (2018). Factor VIII products and inhibitor development in previously treated patients with severe or moderately severe hemophilia A: A systematic review. J. Thromb. Haemost. JTH.

[61] Fischer K., Lassila R., Peyvandi F., Calizzani G., Gatt A., Lambert T., Participants EU HA S.S. (2015). Inhibitor development in haemophilia according to concentrate. Four-year results from the European HAemophilia Safety Surveillance (EUHASS) project. Thromb. Haemost..

[62] Darby S.C., Keeling D.M., Spooner R.J., Wan Kan S., Giangrande P.L., Collins P.W. (2004). The incidence of factor VIII and factor IX inhibitors in the hemophilia population of the UK and their effect on subsequent mortality, 1977–1999. J. Thromb. Haemost. JTH.

[63] Srivastava A., Santagostino E., Dougall A., Kitchen S., Sutherland M., Pipe S.W., Llinás A. (2020). WFH Guidelines for the Management of Hemophilia, 3rd edition. Haemoph. Off. J. World Fed. Hemoph..

